# Denture-associated oral microbiome in dentate and edentulous older adults living in long-term care facilities

**DOI:** 10.1080/20002297.2026.2641915

**Published:** 2026-03-12

**Authors:** Muhammed Manzoor, Pirkko J Pussinen, Riitta KT Saarela, Kaisu Pitkälä, Kaija Hiltunen, Päivi Mäntylä

**Affiliations:** a Institute of Dentistry, University of Eastern Finland, Kuopio, Finland; b Department of Oral and Maxillofacial Diseases, University of Helsinki, Helsinki, Finland; c Department of General Practice and Primary Health Care, University of Helsinki, Helsinki, Finland; d Social Services, Health Care and Rescue Services Division, Oral Health Care, City of Helsinki, Helsinki, Finland; e Unit of Primary Health Care, Helsinki University Hospital, Helsinki, Finland; f Oral and Maxillofacial Diseases, Kuopio University Hospital, Kuopio, Finland

**Keywords:** Oral-systemic diseases (s), edentulous/edentulism, prosthetic dentistry/fixed and removable prosthodontics, microbial ecology, plaque/plaque biofilms, microbiota, dysbiosis

## Abstract

**Background:**

The denture-associated oral microbiome (DAOM) may act as reservoirs of pathogenic microorganisms with potential health effects.

**Objectives:**

To characterize the compositional and functional activity of the DAOM in dentate and edentulous older adults residing in long-term care facilities (LTCFs).

**Methods:**

Participants (51 dentate and 56 edentulous) aged ≥64 years were recruited from the Finnish Oral Health Studies in Older Adults. Clinical oral examinations were performed, and biofilm samples for shotgun metagenomics were collected from the acrylic surface of removable dentures. Diversity indices, taxonomic composition, and functional pathways were assessed to characterize DAOM.

**Results:**

Alpha diversity was similar, whereas beta diversity showed modest differences between groups. Dentate participants had a higher abundance of *Streptococcus mutans*, *Veillonella parvula*, and *Parascardovia denticolens*, whereas edentulous participants were enriched with *Haemophilus parainfluenzae* and *Propionibacterium acidifaciens*. Edentulous participants had reduced microbial network stability and interconnectedness but highly active microbial metabolic functions, particularly those associated with *Streptococcus pneumoniae*.

**Conclusion:**

Although tooth loss does not markedly alter the overall microbial diversity of DAOM, it is associated with distinct taxonomic and functional shifts. Edentulous individuals have less stable and less interconnected microbial networks alongside heightened metabolic activity, reflecting notable changes in the DAOM of older adults living in LTCFs.

## Introduction

A significant proportion of the older population rely on complete or partial removable dentures due to tooth loss, with the prevalence of removable dental prostheses among adults in Europe estimated at 13–29% [1]. The oral environment is inherently complex and shaped by numerous local and external factors, and the introduction of a removable denture adds further ecological complexity. Both natural teeth and denture surfaces serve as substrates for microbial colonisation. Although biofilm formation occurs on both surfaces, denture materials are less exposed to host immune defences, potentially supporting the development of a compositionally distinct plaque microcosm [2]. Inadequate oral and denture hygiene can promote oral dysbiosis and facilitate transition of commensal microorganisms into pathogenic states [3].

The oral environment may shift from health to disease following the use of removable partial dentures [4]. A recent systematic review and meta-analysis encompassing diverse clinical and microbiological methodologies reported significant differences in the oral microbiome of edentulous versus dentate individuals [5]. Among older adults, both alpha and beta diversities of the salivary microbiome differ markedly in edentulous individuals, although age remains a dominant determinant even after adjustment for confounders [6]. The presence of natural dentition profoundly shapes the oral microbiome of denture wearers; even a single remaining tooth can exert a notable impact [2]. The higher microbial diversity observed in dentate individuals extends beyond the teeth to the entire oral cavity [2]. Overall, dentate individuals exhibit a richer and more diverse oral microbiome with declining diversity as tooth loss progresses, although a substantial taxonomic overlap persists [7].

High‑throughput whole‑metagenomic 2bRAD‑M sequencing did not reveal significant differences in taxonomic profiles between denture wearers with active periodontitis and those with a stable periodontal condition [8], although the small sample size may limit these findings. Using the same method, several opportunistic respiratory pathogens have been detected on removable prostheses, with a significantly higher prevalence on unclean dentures than on clean ones [9]. This underscores the potential systemic health implications of poor denture hygiene.

Denture surfaces can harbour complex biofilms containing systemic pathogens, such as *Pseudomonas aeruginosa*, *Streptococcus pneumoniae*, and *Candida* species [8,9]. Consequently, denture-related microbial alterations may contribute to broader systemic conditions, including diabetes, cardiovascular disease, and respiratory infections [5,10,11]. *Candida* species, particularly *C. albicans* and *C. glabrata*, are implicated in chronic low-grade inflammation, insulin resistance, and diabetes progression [12]. Denture use is also linked to an increased risk of cardiometabolic diseases [13]. Periodontal pathogens, such as *Porphyromonas gingivalis* and *Fusobacterium nucleatum*, are frequently detected in denture biofilms and are linked to cardiovascular disease, endothelial dysfunction, and atherosclerosis [14]. Perturbations in the denture-associated oral microbiome (DAOM), identified through 16S rRNA sequencing and targeted PCR for *S. pneumoniae*, are associated with pneumonia in affected patients compared with controls [15]. Aspiration of oral pathogens from denture biofilms is associated with an increased risk of aspiration pneumonia, particularly among older adults and immunocompromised individuals [16,17], and removable denture use is a risk factor for pneumonia in older adults [18]. Conversely, good oral and denture hygiene is associated with a lower risk of aspiration pneumonia in elderly inpatients [19]. DAOM composition may also influence oral infections; our previous study demonstrated marked microbial alterations in denture stomatitis among dentate older participants [20].

Prior studies on denture biofilms have relied on 16S rRNA sequencing or targeted PCR approaches [2,15], which are limited to bacterial profiling. Shotgun metagenomic sequencing permits comprehensive characterisation of bacterial and non-bacterial community members, including archaea and fungi, and their functional metabolic potential, offering substantially deeper insights than traditional methods. Functional profiling further elucidates the biochemical capabilities and ecological behaviour of the microbiome, complementing taxonomic analyses. To our knowledge, our previous study [20] and the present investigation are the first to apply shotgun metagenomics to characterise DAOM in older adults residing in long-term care facilities (LTCFs).

Studying the DAOM in LTCF residents is particularly important because this population is highly susceptible to oral and systemic infections due to advanced age, comorbidities, immunosenescence, and reliance on assisted oral care, which often results in inadequate hygiene. Dentures provide a unique ecological niche that promotes biofilm accumulation and the persistence of opportunistic and respiratory pathogens [9]. This study aimed to comprehensively characterise the taxonomic composition, functional metabolic pathways, and microbial co-occurrence networks of DAOM and to examine how these features differ by dentate status.

## Materials and methods

### Study design and participants

This cross-sectional observational study used data from the Finnish Oral Health Studies in Older Adults (FINORAL), a sub-study involving randomly selected participants from a previous nutrition study (*n *= 550) [21]. Participants were aged ≥64 years and resided in LTCFs in Helsinki, Finland. Individuals who required prophylactic antibiotics, had major deficiencies or refused clinical examination, or died between the completion of the nutrition study and the start of FINORAL were excluded. This resulted in a final sample of 393 subjects who participated in oral examinations [22].

**Figure 1.**
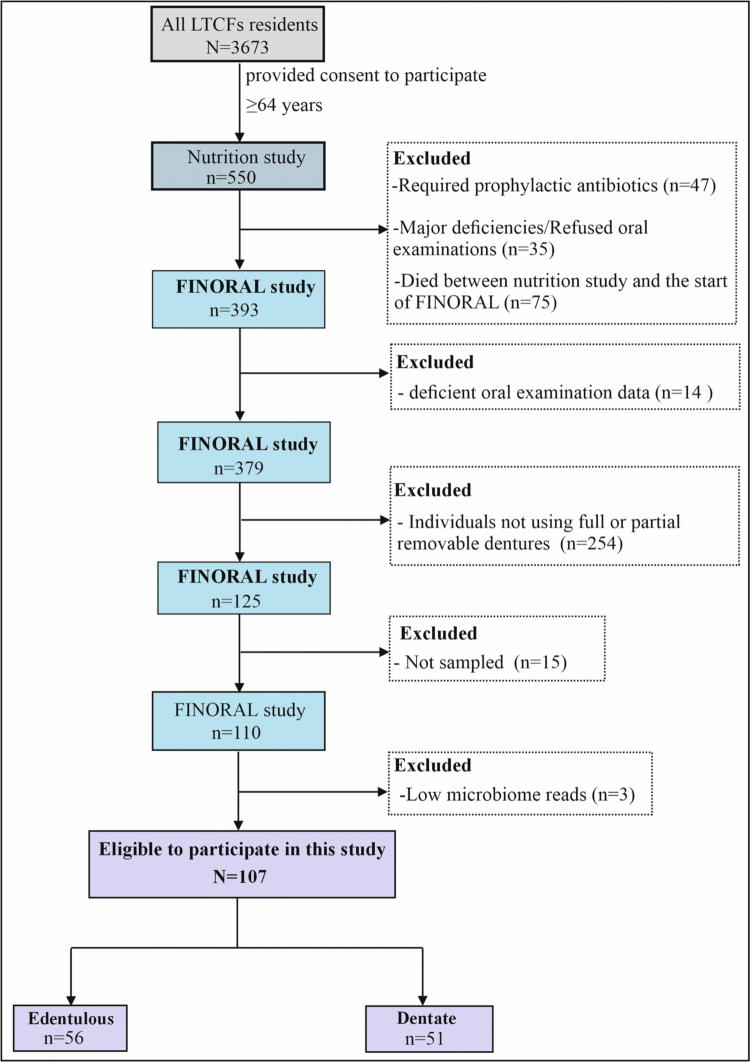
Flowchart of participant selection and exclusions for this study.

Participants who wore full or partial removable dentures and had sufficient biofilm samples for shotgun metagenomic analysis were included in this study. This yielded a final study population of 107 participants (Figure 1).

### Clinical oral examination

The head nurse of the department completed a questionnaire on the demographic characteristics (age, sex, education, diet) of the study participants. Information on antibiotic use during the year prior to the examination and medical diagnoses were obtained from medical records. All participants underwent a comprehensive clinical oral examination conducted by two qualified dentists [22,23]. Examiner calibration was performed using duplicate assessments of the first 10 participants with a consensus agreement. Examinations were performed either at the bedside or while the participant was seated on a chair, depending on the individual’s condition and mobility.

Removable dentures in use were: partial (one or both jaws), complete (one or both jaws), or both types in use. Denture conditions were evaluated and classified as usable, in need of minor repair, in need of major repair, or unusable. Information on denture type, condition, and cleaning practices and sample collection conditions were recorded. Denture stomatitis was evaluated using Newton classification (Type 1, localised inflammation or pinpoint hyperaemia; Type 2, more diffuse erythema [redness] involving part or all of the mucosa that is covered by the denture; and Type 3, inflammatory nodular/papillary hyperplasia) [24]. In addition, the oral mucosa was examined and categorised as healthy, with lesions unrelated to denture use, or with lesions related to denture use. A clinical estimation of oral wetness was recorded to assess oral dryness. Oral wetness was classified as normal salivation (all oral surfaces moist; dental mirror does not stick to the mucosa), somewhat dry mouth (shiny and strained-appearing mucosa with slimy or foam-like saliva), or dry mouth (mirror sticking to the mucosa or tongue, foam-like saliva, glassy mucosal appearance, and fissured or depapillated tongue) [25]. Cognition was assessed based on the participant’s ability to establish and maintain contact during the oral examination and was classified as follows: normal/near normal (established contact, responded appropriately, and supported examination progress), weakened (partial contact with limited responsiveness and contribution), or very weak (no contact, no response, and no assistance with the examination).

The clinical oral examination of dentate participants included recording the number of natural teeth and root remnants; assessment of bacterial plaque accumulation on tooth surfaces (plaque index, PI) and gingival inflammation (gingival index, GI), both registered as the highest score per tooth; bleeding on probing (BOP, recorded as yes/no for each tooth); evaluation of tooth mobility in static mode; pocket probing depth (PPD) measurements at six sites per existing tooth; and documentation of caries lesions [22,26].

Finally, the participants were categorised as dentate (at least one visible tooth or root present) or edentulous (complete edentulism). The exposures were dentate status (dentate vs. edentulous) and denture type (partial, complete, or both). The primary outcomes were microbiome diversity, taxonomic composition, and functional pathways. Potential confounders included age, sex, cognitive status, medications, oral hygiene practices, and denture condition.

### Sample collection, DNA isolation, and sequencing

After intraoral examination, a dentist collected the biofilm from the pink acrylic fitting surface of the removable denture using a curette. Scratching was performed for approximately 5 sec to ensure adequate biofilm collection. The material was pooled into 1.5-mL microcentrifuge tubes containing PCR-grade water, initially stored at −20 °C and later transferred to −80 °C until DNA extraction. Standard microbiological procedures were followed to minimise the risk of microbial contamination during sample collection and processing. A total of 500 µL of the microbial sample was combined with 500 µL of lysis buffer in NucleoSpin Bead Tubes Type B (Macherey-Nagel). Metagenomic DNA was purified using the Chemagic DNA Blood 400-H96 Kit, optimised for the Chemagic™ 360 instrument (PerkinElmer), with the Chemagic Saliva600 pre-filled protocol following the manufacturer’s instructions. DNA was eluted in elution buffer, and concentration was quantified fluorometrically using the Qubit dsDNA Broad Range Assay Kit (Invitrogen, Carlsbad, CA, USA) on a DeNovix DS-11 FX+ Fluorometer (DeNovix Inc., Wilmington, DE, USA).

Libraries for next-generation sequencing were prepared using the NEBNext® Ultra™ II FS DNA Library Prep Kit. Shotgun sequencing was performed using an Illumina NovaSeq 6000 sequencing platform (Illumina, USA) using 150-bp paired-end reads. Metagenomic raw sequence quality was assessed using FastQC (v0.12.1) and MultiQC (v1.14). Adaptor sequences and low-quality bases were removed with Trimmomatic (v0.39). Host-associated reads were removed using KneadData (v0.12.0) with the default human reference genome (hg37). Processed reads were then mapped to a reference database using Kraken2 (v2.1.3) [27] for taxonomic classification. Species-level abundance estimation was performed using Bracken (v3.0) [28]. Methods for taxonomic annotation have been described previously [29,30].

### Statistical analyses

Statistical analyses were performed using R (v. 4.5.1). Descriptive statistics were computed and reported as median and interquartile range (IQR) for continuous variables and compared using the Mann-Whitney *U*-test. Categorical variables are presented as absolute numbers and percentages and were compared using the Pearson χ² test or Fisher’s exact test.

Before microbiome analysis, samples and taxa with zero counts or <1000 reads were excluded. The remaining samples were rarefied, and taxa present in <25% of the samples were excluded. Alpha diversity metrics (observed richness and Shannon, Inverse Simpson, and Fisher indices) were calculated using the *phyloseq* package (v1.52.0) in R and group-wise comparisons were performed using the Wilcoxon rank-sum test. Beta diversity was assessed using the Bray-Curtis and Jaccard dissimilarity metrics, and differences were tested using permutational multivariate analysis of variance (PERMANOVA) in the *vegan* R package (v.2.7.2) based on 999 permutations. Models were adjusted for age, gender, diabetes, smoking status, residency in the current facility, diet, dementia, denture stomatitis, and oral mucosal status.

Linear Discriminant Analysis Effect Size (LEfSe) [31] was used to identify the differentially abundant taxa between edentulous and dentate participants. Taxa were considered significant at Kruskal-Wallis *p *< 0.05 and an LDA score >3 to focus on taxa with stronger discriminatory effects and to reduce the inclusion of taxa with minimal biological relevance. Spearman’s rank correlation coefficients (*ρ*) were computed between microbial abundances and clinical and oral variables. HUMAnN3 was used to profile the abundance of microbial functional pathways [32]. Differentially abundant pathways between the edentulous and dentate participants were assessed using MaAsLin2 (v1.22.0) in R [33]. Microbial co-occurrence networks were constructed using the SpiecEasi package in R (v1.99.0), and the network topological metrics were subsequently computed.

## Results

### Characteristics of the study participants

A total of 107 participants (56 edentulous and 51 dentate) met the inclusion criteria and were included in the study (Figure 1). Clinical characteristics of the participants are presented in Table 1. The median (IQR) age was similar between groups (87 [8] years); 78 (72.9%) participants were female. Median (IQR) length of stay at the current facility was 30 (41) months in both groups. Edentulous participants ate soft food more often than dentate participants (37.3% vs. 17%, *p *= 0.025).

**Table 1.** Characteristics of the study participants.

	Edentulous (*n *= 56)	Dentate (*n *= 51)	All (*n *= 107)	*p*-value
	**Median (IQR)**			
Age, years	86.5 (9)	87 (8)	87 (8)	0.955
Residency in current facility, months	34 (39)	24 (41)	30 (41)	0.445
	***n* (%)**			
Sex, female	45 (80.4)	33 (64.7)	78 (72.9)	0.069
**Smoking**				0.636
Never	23 (62.2)	21 (56.8)	44 (59.5)	
Previous	14 (37.8)	16 (43.2)	30 (40.5)	
Diabetes, yes	7 (13.5)	11 (22.4)	18 (17.8)	0.238
**Daily medications, months**				0.985
≤5	10 (19.6)	9 (18.4)	19 (19)	
≥6	41 (80.4)	40 (81.6)	81 (81)	
Antibiotic use during last year, yes	25 (53.2)	20 (48.8)	45 (51.1)	0.680
**Dementia**				0.193
No or mild	13 (26.5)	19 (40.4)	32 (33.3)	
Moderate or severe	36 (73.5)	28 (59.6)	64 (66.7)	
**Contact ability/cognition during oral examination**				0.134
Normal/near normal	29 (52.7)	36 (72)	65 (61.9)	
Weakened	17 (30.9)	10 (20)	27 (25.7)	
Very weak	9 (16.4)	4 (8)	13 (12.4)	
**Diet**				0.025
Ordinary	32 (62.7)	39 (83)	71 (72.4)	
Soft	19 (37.3)	8 (17)	27 (27.6)	
**Dryness of mouth**				0.920
No signs of dryness/normal	11 (21.2)	12 (24)	23 (22.5)	
Somewhat dry	26 (50)	25 (50)	51 (50)	
Dry	15 (28.8)	13 (26)	28 (27.5)	
**Oral mucosa**				0.030
Healthy	42 (75)	26 (51)	68 (63.6)	
Lesion not related to dentures	2 (3.6)	2 (3.9)	4 (3.7)	
Lesion related to dentures	12 (21.4)	23 (45.1)	35 (32.7)	
Denture stomatitis, yes	10 (17.9)	20 (39.2)	30 (28)	0.014
**Denture cleaning**				0.224
By participant	11 (23.9)	16 (35.6)	27 (29.7)	
Someone else	35 (76.1)	29 (64.4)	64 (70.3)	
Denture use at night, yes	18 (37.5)	18 (39.1)	36 (38.3)	0.871
**Denture type**				<0.001
Partial (1-2 jaws)	1 (1.9)	11 (21.6)	12 (11.4)	
Complete (1-2 jaws)	52 (96.3)	29 (59.9)	81 (77.1)	
Both	1 (1.9)	11 (21.6)	12 (11.4)	
**Denture condition**				0.601
No need of repair	31 (57.4)	30 (62.5)	61 (59.8)	
In need of repair	23 (42.6)	18 (37.5)	41 (40.2)	

No statistically significant differences were observed in smoking, diabetes, number of medications, antibiotic use during the last year, cognitive function, and dementia status between the edentulous and dentate groups. Denture stomatitis (39.2% vs. 17.9%, *p *= 0.014) and mucosal lesions (45.1% vs. 21.4%, *p *= 0.030) were more common among dentate than edentulous participants. No significant differences were found in denture cleaning, night-time denture wearing, or denture conditions between groups. Among participants, 11.4% used partial dentures, 77.1% used complete dentures, and 11.4% used both types of dentures, with a significant difference in denture type distribution between the groups (*p *< 0.001). The dentate participants had a mean (median) number of 6.8 (7) teeth. Clinical findings are shown in Table S1.

### Composition and diversity of the DAOM in dentate and edentulous participants

**Figure 2.**
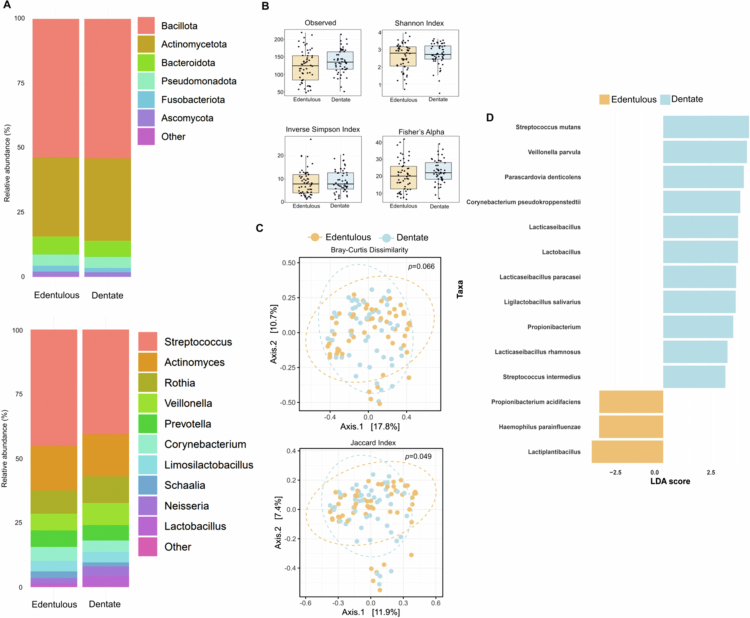
Denture-associated oral microbiome in dentate and edentulous participants. (A) Stacked bar plots showing the mean relative abundance of microbial taxa at the phylum and genus levels. Taxa are ordered from top to bottom by decreasing relative abundance in dentate and edentulous groups. Only the most prevalent taxa are shown; others are grouped as “Other.” (B) Boxplots showing differences in alpha diversity of denture-associated oral microbiome (DAOM) at the species level between dentate and edentulous participants. (C) Beta diversity of DAOM shown by non-metric multidimensional scaling (nMDS) based on Bray-Curtis dissimilarity. Each dot represents a sample; distances reflect differences in overall microbial composition. Ellipses indicate 95% confidence intervals for each group. (D) Differential abundance of DAOM between dentate and edentulous participants. Bars show log-fold changes for each taxon; colours indicate enrichment in either dentate or edentulous participants.

The DAOM was dominated by Bacillota, Actinomycetota, Bacteroidota, Pseudomonadota, Fusobacteriota, Ascomycota, and Campylobacterota, which accounted for >98% of the total relative abundance across all samples (Figure 2A). Among these, Bacillota (~53%), Actinomycetota (~31%), and Bacteroidota (~6%) predominated in both dentate and edentulous participants, with slightly higher abundances of Fusobacteriota and Campylobacterota in edentulous participants (Table S2). At the genus level, *Streptococcus*, *Actinomyces*, *Prevotella*, *Corynebacterium*, and *Schaalia* were more abundant in edentulous participants, whereas *Veillonella*, *Rothia*, *Neisseria*, and *Lactobacillus* were relatively less abundant (Figure 2A; Table S2).

None of the alpha diversity metrics showed significant differences in richness or evenness between dentate and edentulous participants. Observed richness was 124.5 (IQR 69.5) in edentulous and 135 (IQR 50) in dentate individuals. Similarly, Shannon diversity (2.8 [1.1] vs. 2.7 [0.75]), inverse Simpson index (7.85 [7.9] vs. 7.83 [7.03]), and Fisher’s index (20.1 [13.4] vs. 22.1 [9.8]) did not differ between groups (Figure 1B). PERMANOVA (adonis) analyses indicated that none of the demographic or clinical variables significantly explained the differences in microbial community composition (Table S3). PCoA plots (Figure 2C) and PERMANOVA results comparing edentulous and dentate participants revealed modest differences in the unadjusted models (Bray-Curtis: R² = 0.0148, *p *= 0.066; Jaccard: R² = 0.0137, *p *= 0.049), which were however attenuated after adding diabetes, smoking, and type of diet in multivariate models (Table S4).

LEfSe analysis identified 14 taxa that differed between the dentate and edentulous participants. Among these, 11 taxa were significantly more abundant in dentate participants (Figure 2D), with *Streptococcus mutans* showing the largest difference (LDA = 4.49, *p *= 0.001), followed by *Veillonella parvula* (LDA = 4.38, *p *= 0.010) and *Parascardovia denticolens* (LDA = 4.22, *p *= 0.010). Edentulous participants were enriched with *Haemophilus parainfluenzae* (LDA = 3.36, *p *= 0.039) and *Propionibacterium acidifaciens* (LDA = 3.35, *p *= 0.023).

### DAOM taxa correlated with clinical phenotypes

**Figure 3.**
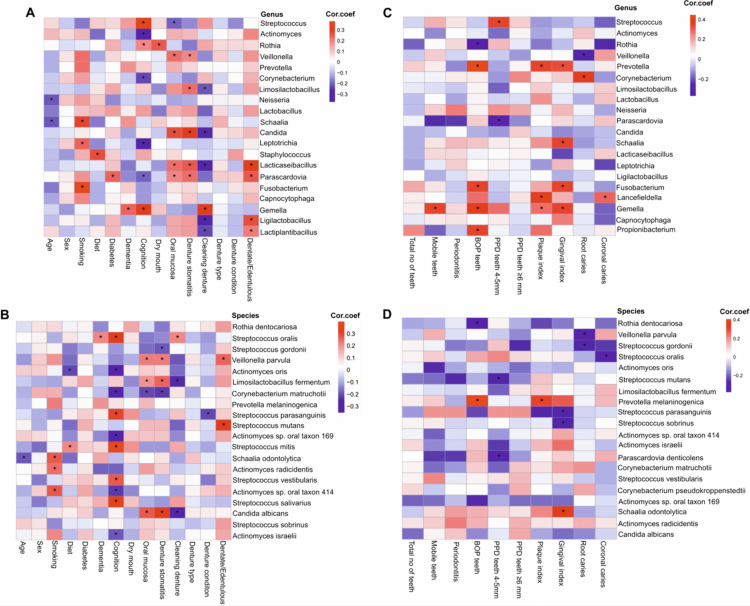
Heatmap showing Spearman correlation coefficients between top taxa and clinical or oral health variables. The top 20 most abundant (A) genera and (B) species in all participants, and the top 20 most abundant (C) genera and (D) species in dentate participants only. Correlation coefficients are shown within each cell, and significant correlations (*P *< 0.05) are marked with an asterisk (*). Blue indicates negative correlations; red indicates positive correlations. Rows and columns are hierarchically clustered.

Correlation analysis (Figure 3) between oral genera and dentate status revealed strong positive associations with *Lacticaseibacillus*, *Parascardovia*, *Ligilactobacillus*, and *Lactiplantibacillus*. Other genera showed weaker or non-significant correlations with denture status, type, and condition (Figure 3A). Lifestyle and clinical factors further influenced the genus-level composition. *Fusobacterium* and *Leptotrichia* were positively associated with smoking, whereas *Streptococcus* and *Gemella* were positively correlated with declining cognition. Similarly, species-level analysis revealed several significant correlations (Figure 3B). For example, *S. mutans* and *Veillonella parvula* were positively associated with dentate status, whereas multiple *Actinomyces* species, including *A. oris*, *A. israelii*, and *Actinomyces* sp. oral taxon 414 were negatively associated with cognitive function. The *Candida* genus and its species *C. albicans* were positively correlated with denture stomatitis and oral mucosal changes but negatively correlated with denture cleaning frequency (Table S5).

In dentate participants, *Prevotella* and *Gemella* were positively correlated with BOP, PI, and GI (Figure 3C), whereas several *Streptococcus* species were negatively correlated with these parameters. In particular, *S. mutans* was negatively associated with PPD, whereas *S. parasanguinis* and *S. sobrinus* were negatively associated with GI and *S. gordonii* with root caries (Figure 3D; Table S6).

### Microbial co-occurrence networks in edentulous and dentate participants

**Figure 4.**
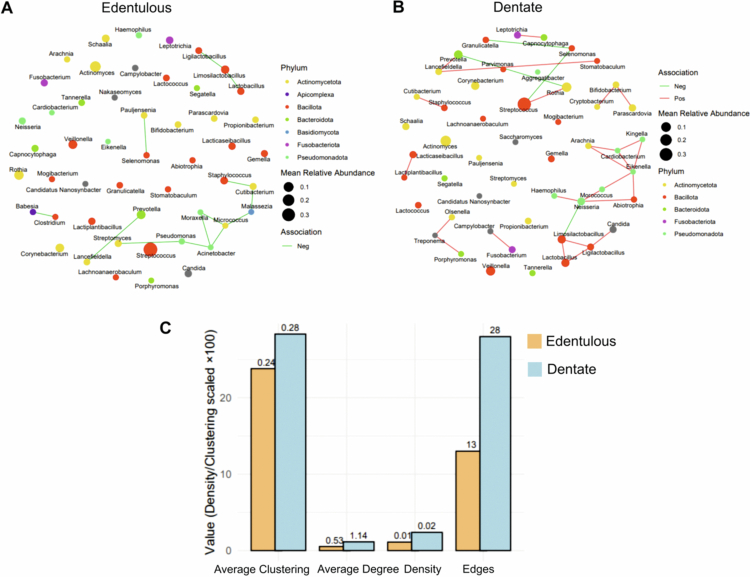
Co-occurrence networks and network topography metrics of the top denture-associated oral microbiome in edentate and dentate participants. Co-occurrence networks for the most abundant genera in (A) edentulous and (B) dentate participants. Nodes represent genera, coloured by phylum, with node size proportional to mean relative abundance across samples. Edges represent significant associations between genera, coloured dark green for negative correlations and red for positive correlations. (C) Bar plot showing network topography metrics for edentulous and dentate participants.

Co-occurrence network analysis revealed distinct microbial interaction patterns between edentulous and dentate participants (Figure 4A,B). The DAOM of dentate participants formed a more connected and structured network compared with that of edentulous participants. The average clustering coefficient was also higher in dentate participants (0.28 vs. 0.24; Figure 4C), indicating a more interconnected community structure. Hub genera in the edentulous network included *Micrococcus*, *Acinetobacter*, *Limosilactobacillus*, *Cutibacterium*, and *Pseudomonas*, whereas *Eikenella*, *Neisseria*, *Ligilactobacillus*, *Limosilactobacillus*, and *Selenomonas* were identified as central taxa in dentate participants (Table S7).

### Functional pathway differences between edentulous and dentate DAOM

**Figure 5.**
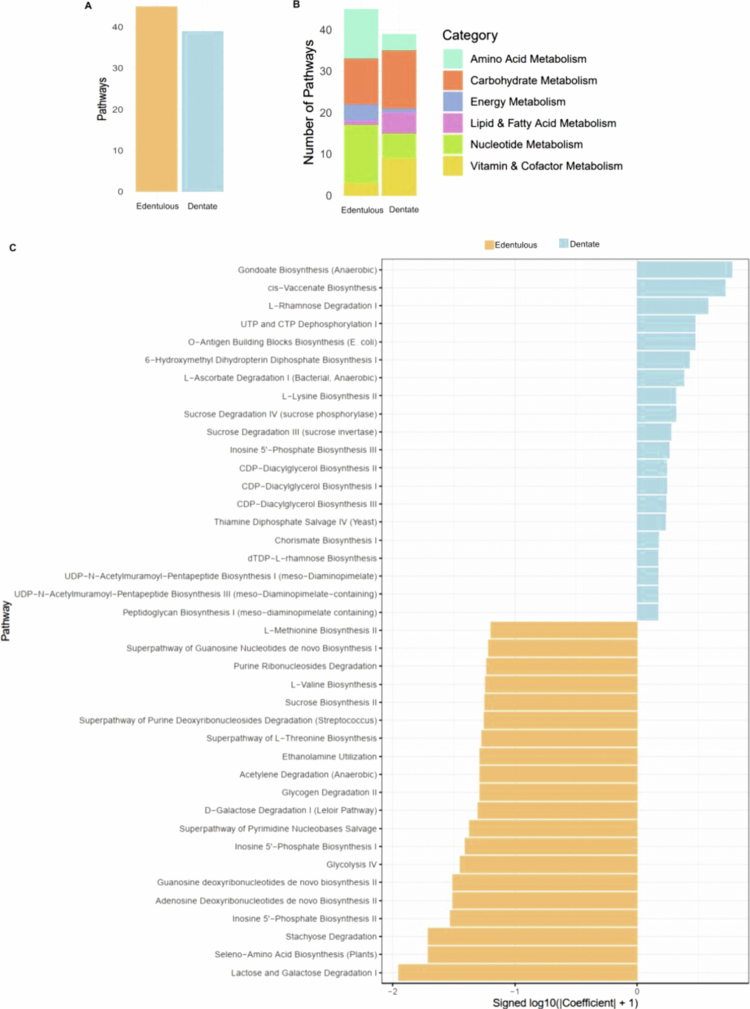
Functional profiling of denture-associated oral microbiome in dentate and edentulous participants. (A) Bar plot showing the number of significant pathways associated with dentate and edentulous participants. (B) Number of pathways categorised based on their functional category. (C) Bar plot showing the list of top 40 significant pathways associated with dentate and edentulous participants.

Functional analysis revealed 84 significantly enriched pathways (Figure 5A). In edentulous participants, DAOM showed increased activity in nucleotide, amino acid, and energy metabolism pathways, whereas carbohydrate and vitamin/cofactor metabolism predominated in dentate participants (Figure 5B). The markedly enriched pathways in dentate participants also included gondoate and cis-vaccenate and O-antigen building blocks. In contrast, several pathways were reduced in the DAOM of edentulous participants, including lactose and galactose degradation, inosine 5′-phosphate biosynthesis II, and adenosine and guanosine deoxyribonucleotide de novo biosynthesis (Figure 5C and Table S8).

## Discussion

Our metagenomics analysis of removable denture biofilms revealed clear differences in the DAOM between dentate and edentulous participants. These differences encompassed (i) distinct microbial community structures, (ii) differential abundance of four genera and 10 species, (iii) reduced microbial network stability and connectedness in edentulous individuals, and (iv) heightened metabolic activity despite lower network complexity. Together, these findings indicate that edentulism not only reshapes the taxonomic composition of the DAOM but also drives substantial functional reorganisation.

Denture surfaces can harbour more than 10¹¹ microbial cells/mg [34], contributing to denture stomatitis, oral candidiasis, and other oral infections. Previous 16S rRNA studies have shown that dental and denture plaques differ in composition and diversity, with denture and mucosal sites predominantly colonised by Bacilli and Actinobacteria [2]. In our study, alpha diversity did not differ significantly between dentate and edentulous participants, suggesting comparable overall microbial richness and evenness. Although salivary bacterial diversity is typically lower in edentulous individuals [7], the microbial load on denture surfaces appears similar across groups. While denture and tooth surfaces within an individual share similar bacterial communities [35], the presence or absence of natural teeth was the strongest determinant of DAOM composition in our cohort. Other covariates, including age, sex, smoking, diet, dementia, diabetes, and oral diseases had only minor effects.

At the phylum level, edentulous participants exhibited higher relative abundances of Bacteroidota, Fusobacteriota, and Campylobacterota, whereas other phyla were comparable between groups. Dentate participants showed greater abundance of typical oral commensals such as *Veillonella*, *Rothia*, *Neisseria*, and *Lactobacillus*, while edentulous participants were enriched with *Streptococcus*, *Actinomyces*, *Prevotella*, *Corynebacterium*, and *Schaalia*. These patterns differ somewhat from earlier findings reporting higher *Actinomyces* and *Corynebacterium* in dentate individuals and higher *Lactobacillus* in edentulous adults [2]. A high proportion of *Veillonella* is associated with both complete dentures and denture wear with remaining teeth [2,7,35]. Consistent with our results, a meta-analysis reported markedly lower *Veillonella* abundance in complete denture wearers, suggesting that natural teeth provide a favourable niche for this genus [5].

Co-occurrence network analysis further highlighted ecological restructuring. In edentulous participants, hub genera included *Micrococcus*, *Acinetobacter*, *Cutibacterium*, and *Pseudomonas*, whereas dentate participants were characterised by hubs such as *Eikenella*, *Neisseria*, and *Selenomonas*. Members of the Lactobacillaceae family formed important hubs in both groups. These hubs were not necessarily the most abundant taxa but exerted disproportionate influence on community structure. The shift from typical oral commensals in dentate individuals to more environmental taxa in edentulous individuals [36] likely reflects changes in habitat and host factors. Differences from earlier systematic reviews, where *Candida*, *Streptococcus*, and *Porphyromonas* were highly connected in complete denture wearers [5], may stem from methodological variation, as prior studies relied on 16S rRNA sequencing, targeted PCR, culture-based approaches, and case-control designs including denture stomatitis. Indeed, *Ascomycota* forms a central hub in denture stomatitis, interacting strongly with Bacillota [20].

Cognitive decline correlated particularly with *Gemella* and *Streptococcus* in the DAOM, which is consistent with some previous studies [37,38] but contrasts with a systematic review that included heterogeneous analytical methods [39]. *Candida*, *Veillonella*, *Lacticaselbacillus*, and *Parascardovia* were strongly associated with denture stomatitis and mucosal lesions, supporting the role of bacterial-fungal interactions in biofilm pathogenicity [20,40]. In dentate participants, *Prevotella*, *Fusobacterium*, *Lancefieldella*, and *Gemella* correlated with BOP, PI, and GI, consistent with their known associations with oral disease [41]. Notably, DAOM species did not correlate with periodontal pocket depth, suggesting that microbial shifts in denture biofilms may occur independently of traditional periodontal indicators.

Functional profiling revealed pronounced metabolic differences between groups. In edentulous participants, DAOM showed increased activity in nucleotide, amino acid, and energy metabolism pathways, indicating elevated biosynthetic and energetic demands. In contrast, pathways related to carbohydrate, lipid and fatty acid, and vitamin and cofactor metabolism were reduced. Decreased activity in lactose and galactose degradation, inosine 5′-phosphate biosynthesis II, and deoxyribonucleotide biosynthesis suggests reduced carbohydrate utilisation and lower nucleotide demand, potentially reflecting slower microbial growth [42]. These patterns point to a streamlined metabolic profile favouring energy conservation and survival under altered ecological conditions. In dentate participants, several metabolic pathways provided insight into microbial adaptation within denture biofilms. Gondoate and cis-vaccenate biosynthesis, which is key for unsaturated fatty acid production, may enhance membrane stability and stress tolerance [43]. L-rhamnose degradation supports utilisation of host-derived glycoproteins [44], while O-antigen building block biosynthesis contributes to lipopolysaccharide formation and bacterial virulence [45]. Lipopolysaccharide biosynthesis was especially addressed to *F. nucleatum*, which along with genus *Fusobacterium* associated directly with dentate status and number of teeth.

Denture biofilms serve as reservoirs for pro-inflammatory and pathogenic microorganisms that contribute to chronic inflammation [2] and systemic conditions, including diabetes, cardiovascular disease, and respiratory infections [10,11]. Of note, 22/84 (26.2%) metabolic pathways associated with edentulism were linked to the metabolism of *S. pneumoniae*, which can cause invasive infections of the brain, sepsis, or pneumonia. Understanding how denture use shapes microbial ecology may therefore have important implications for both oral and systemic health in older adults.

The denture microbiome exhibits resilience to hygiene interventions. *In vitro* studies show that *C. albicans* is largely unresponsive to cleansing, whereas bacterial biofilms can repopulate rapidly yet remain susceptible to repeated cleaning [46]. Denture material, design, age, storage method, and duration of use also influence microbiome composition [5,47,48]. Suboptimal prosthetic manufacturing may contribute to pathological conditions [49]. Although advances in materials science aim to develop prosthetic bases with anti-adhesive or antimicrobial properties [50], the presence of a removable denture alone can disrupt oral microbial balance regardless of material [51].

The strengths of this study include detailed clinical oral examinations, closely matched participant groups in terms of characteristics, data extraction from medical records, and use of shotgun metagenomics. The study population, namely very old adults living in LTCFs, is a population that typically uses multiple medications, is functionally impaired, and is particularly vulnerable to oral health complications and frailty. Limitations included the cross-sectional design, sampling conditions that were adapted to LTCF routines, and participant instructions (e.g. wearing dentures for a set duration, avoiding eating, drinking, or cleaning beforehand) that could not be strictly enforced. Selection bias was related to the recruitment of participants from LTCFs. Although denture type, condition, cleaning practices, and sampling procedures were recorded and included as covariates in the analyses, residual variation may have influenced the microbial profiles. Maintaining appropriate oral hygiene, including regular mechanical cleaning, overnight storage in water with a cleansing tablet, and periodic professional maintenance is essential to reduce pathogenic biofilm formation and support oral health in denture wearers [52].

## Conclusion

Shotgun metagenomic analysis of removable denture biofilms revealed marked differences in the DAOM between dentate and edentulous participants. Collectively, the taxonomic, network, and functional divergences observed emphasise the extensive ecological and metabolic reorganisation of the DAOM following tooth loss and denture use. The potential increase in DAOM pathogenicity following tooth loss is a significant concern for older adults living in LTCFs.

## Supplementary Material

Appendix_JOM.docx Appendix_JOM.docx

## Data Availability

The datasets generated and/or analysed during the current study are not publicly available due to licensing restrictions from the City of Helsinki but are available from the corresponding author on reasonable request and with permission from the City of Helsinki.
