# Supplementary material for: Denture-associated oral microbiome in dentate and edentulous older adults living in long-term care facilities
Source: J Oral Microbiol. 2026 Mar 12;18(1):2641915. doi: 10.1080/20002297.2026.2641915 (PMC12983798; doi:10.1080/20002297.2026.2641915)
Supplement: Appendix_JOM.docx [file ZJOM_A_2641915_SM3359.docx]

**Appendix**

**Table S1.** Clinical oral characteristics of dentate participants

|  | median (IQR) | mean (SD) |
| --- | --- | --- |
| N of teeth* | 7 (6) | 6.8 (4.2) |
| Residual roots | 0 (1) | 1.1 (2.2) |
| PPD teeth  4-5 mm  ≥6 mm | 0 (2)  0 (0) | 1.2 (1.9)  0.1 (0.4) |
| Increased mobility | 0 (2) | 0.9 (1.4) |
| BOP % | 100 (46) | 74.9 (39.6) |
| PI | 2.1 (1.3) | 2.2 (1.0) |
| GI | 2 (1) | 1.5 (0.9) |
| Root surface caries | 0 (1) | 0.9 (1.4) |
| Crown caries | 0.2 (0) | 0.2 (0.7) |
|  | **n (%)** |  |
| Clinical diagnosis of periodontitis  Healthy periodontium  Gingivitis  Mild periodontitis Localized advanced periodontitis | 1 (4.8)  24 (57.1)  13 (31)  3 (7.1) |  |

*Total n including also residual roots

**Table S2.** Relative abundance of major DAOM phyla and genera in edentulous and dentate participants (Mean ± SD, %)

|  | **Edentulous** | **Dentate** | **Adjusted *P V*alue** |
| --- | --- | --- | --- |
|  | **Phylum** |  |  |
| Bacillota | 53.2 ± 33.6 | 53.9 ± 27.5 | 0.962 |
| Actinomycetota | 30.3 ± 28.3 | 31.8 ± 25.3 | 0.891 |
| Bacteroidota | 6.8 ± 11.7 | 6.1 ± 9.1 | 0.890 |
| Pseudomonadota | 4.26 ± 10.80 | 4.26 ± 11.13 | 0.752 |
| Fusobacteriota | 2.42 ± 4.44 | 1.74 ± 2.97 | 0.891 |
| Ascomycota | 1.8 ± 5.6 | 1.75 ± 5.1 | 0.891 |
| Campylobacterota | 0.38 ± 0.67 | 0.18 ± 0.31 | 0.890 |
|  | **Genus** |  |  |
| *Streptococcus* | 36.9 ± 33.2 | 34.7 ± 25.1 | 0.972 |
| *Actinomyces* | 14.1 ± 22.4 | 13.9 ± 18.6 | 0.190 |
| *Rothia* | 7.4 ± 15.8 | 8.9 ± 19.0 | 0.614 |
| *Veillonella* | 5.2 ± 6.5 | 7.3 ± 7.4 | 0.190 |
| *Prevotella* | 5.2 ± 11.2 | 5.1 ± 8.6 | 0.614 |
| *Corynebacterium* | 4.3 ± 10.8 | 3.7 ± 10.4 | 0.401 |
| *Limosilactobacillus* | 3.4 ± 13.5 | 3.5 ± 6.5 | 0.190 |
| *Neisseria* | 1.6 ± 4.2 | 3.2 ± 9.7 | 0.972 |
| *Lactobacillus* | 1.0 ± 3.6 | 3.4 ± 8.4 | 0.190 |
| *Schaalia* | 2.1 ± 3.8 | 1.3 ± 2.2 | 0.741 |

PERMANOVA (adonis) results for associations between microbial community

composition (Bray–Curtis and Jaccard) and demographic and/or clinical variables.

|  | **Df** | **SumOfSqs** |  | **R^2^** | **F** | **Pr(>F)** |
| --- | --- | --- | --- | --- | --- | --- |
|  |  | **Bray-Curtis** | |  |  | |
| Dentate/ edentulous | 1 | 0.380 |  | 0.017 | 1.074 | 0.356 |
| Age | 1 | 0.239 |  | 0.011 | 0.676 | 0.843 |
| Sex | 1 | 0.243 |  | 0.011 | 0.686 | 0.826 |
| Diabetes | 1 | 0.269 |  | 0.012 | 0.761 | 0.757 |
| Smoking | 1 | 0.376 |  | 0.017 | 1.061 | 0.378 |
| Diet | 1 | 0.256 |  | 0.012 | 0.723 | 0.772 |
| Dementia | 1 | 0.389 |  | 0.018 | 1.101 | 0.3 |
| Mobility for movement | 1 | 0.223 |  | 0.010 | 0.630 | 0.874 |
| Denture stomatitis | 1 | 0.325 |  | 0.015 | 0.918 | 0.535 |
| Oral mucosal lesions | 1 | 0.278 |  | 0.013 | 0.787 | 0.717 |
| Residency in current facility, months | 1 | 0.372 |  | 0.017 | 1.050 | 0.369 |
| Residual | 53 | 18.778 |  | 0.855 |  |  |
| Total | 64 | 22.13 |  | 1 |  |  |
|  |  | **Jaccard** | |  |  | |
| Dentate/ edentulous | 1 | 0.441 |  | 0.017 | 1.081 | 0.328 |
| Age | 1 | 0.320 |  | 0.012 | 0.783 | 0.846 |
| Sex | 1 | 0.331 |  | 0.012 | 0.810 | 0.788 |
| Diabetes | 1 | 0.347 |  | 0.013 | 0.850 | 0.746 |
| Smoking | 1 | 0.452 |  | 0.017 | 1.108 | 0.275 |
| Diet | 1 | 0.349 |  | 0.013 | 0.854 | 0.720 |
| Dementia | 1 | 0.455 |  | 0.017 | 1.115 | 0.262 |
| Mobility for movement | 1 | 0.295 |  | 0.011 | 0.723 | 0.928 |
| Denture stomatitis | 1 | 0.386 |  | 0.014 | 0,.947 | 0.546 |
| Oral mucosal lesions | 1 | 0.351 |  | 0.013 | 0.860 | 0.704 |
| Residency in current facility, months | 1 | 0.438 |  | 0.017 | 1.073 | 0.340 |
| Residual | 53 | 21.648 |  | 0.838 |  |  |
| Total | 64 | 25.818 |  | 1 |  |  |

42 Beta Diversity (Bray-Curtis and Jaccard) PERMANOVA results comparing 43 edentulous and dentate participants

| **Metric** | **Model** | **R²**  **(Group)** | **F (Group)** | ***P*-value** |
| --- | --- | --- | --- | --- |
| Bray-Curtis | Model 1: edentulous vs. dentate | 0.0148 | 1.5761 | 0.066 |
|  | Model 2: edentulous vs. dentate + Age + Sex | 0.0148 | 1.5703 | 0.072 |
|  | Model 3: edentulous vs. dentate + Diabetes + Smoking + Diet | 0.0192 | 1.3033 | 0.162 |
|  | Model 4: edentulous vs. dentate + Dementia + Mobility + Denture stomatitis + Oral mucosa +  Residency in current facility, months | 0.0172 | 1.0737 | 0.363 |
| Jaccard | Model 1: edentulous vs. dentate | 0.0137 | 1.4614 | 0.049 |
|  | Model 2: edentulous vs. dentate + Age + Sex | 0.0137 | 1.4586 | 0.039 |
|  | Model 3: edentulous vs. dentate + Diabetes + Smoking + Diet | 0.0178 | 1.2170 | 0.168 |
|  | Model 4: edentulous vs. dentate + Dementia + Mobility + Denture stomatitis + Oral mucosa +  Residency in current facility, months | 0.0171 | 1.0817 | 0.318 |

44

45

46

47

1. Spearman correlation coefficients between the top most abundant genera and species and clinical/oral health variables in all
2. participants

| **Genus** | **Dentate/**  **Edentouolous** | **Age** | **Sex** | **Smoking** | **Diet** | **Diabetes** | **Dementia** | **Cognition** |
| --- | --- | --- | --- | --- | --- | --- | --- | --- |
| *Streptococcus* | 0.005 | 0.079 | -0.073 | -0.175 | 0.087 | 0.073 | 0.155 | 0.364 |
| *Actinomyces* | 0.188 | 0.113 | 0.071 | 0.122 | -0.183 | 0.024 | -0.122 | -0.374 |
| *Rothia* | 0.078 | -0.006 | -0.052 | -0.029 | 0.018 | 0.134 | -0.077 | 0.199 |
| *Veillonella* | 0.178 | -0.117 | 0.056 | 0.223 | -0.125 | 0.066 | 0.033 | 0.004 |
| *Prevotella* | 0.086 | -0.104 | 0.004 | 0.218 | -0.048 | 0.016 | 0.170 | 0.083 |
| *Corynebacterium* | 0.125 | -0.086 | 0.086 | 0.163 | -0.039 | 0.027 | -0.072 | -0.223 |
| *Limosilactobacillus* | 0.170 | -0.081 | -0.066 | -0.087 | 0.051 | 0.154 | -0.030 | 0.083 |
| *Neisseria* | -0.012 | -0.213 | 0.103 | 0.169 | 0.081 | -0.160 | -0.064 | -0.083 |
| *Lactobacillus* | 0.167 | -0.169 | -0.010 | 0.098 | 0.115 | 0.160 | 0.040 | 0.155 |
| *Schaalia* | -0.051 | -0.213 | 0.004 | 0.278 | -0.174 | 0.015 | 0.173 | -0.044 |
| *Candida* | 0.124 | -0.067 | -0.035 | 0.035 | 0.012 | 0.088 | -0.120 | 0.037 |
| *Leptotrichia* | 0.040 | -0.072 | 0.081 | 0.232 | -0.160 | -0.033 | -0.075 | -0.282 |
| *Staphylococcus* | 0.018 | -0.101 | -0.133 | 0.129 | 0.262 | 0.086 | -0.070 | 0.143 |
| *Lacticaseibacillus* | 0.314 | -0.102 | -0.056 | -0.032 | -0.030 | 0.104 | -0.165 | -0.037 |
| *Parascardovia* | 0.249 | 0.012 | 0.042 | 0.170 | -0.072 | 0.240 | -0.151 | -0.197 |
| *Fusobacterium* | 0.081 | -0.168 | 0.024 | 0.381 | -0.105 | 0.007 | 0.093 | -0.139 |
| *Capnocytophaga* | 0.124 | -0.126 | 0.073 | 0.198 | -0.115 | 0.032 | 0.033 | -0.144 |
| *Gemella* | -0.093 | -0.064 | -0.136 | -0.025 | 0.014 | 0.029 | 0.243 | 0.316 |
| *Ligilactobacillus* | 0.272 | -0.094 | 0.024 | 0.043 | -0.115 | 0.115 | -0.109 | -0.024 |
| *Lactiplantibacillus* | 0.211 | 0.066 | -0.098 | -0.046 | 0.039 | 0.124 | -0.018 | 0.041 |
| **Genus** | **Dry mouth** | **Oral mucosa** | **Denture stomatitis** | **Cleaning of denture** | **Denture type** | **Denture condition** |  |  |
| *Streptococcus* | 0.078 | -0.200 | -0.156 | 0.175 | -0.064 | -0.151 |  |  |
| *Actinomyces* | 0.016 | 0.139 | 0.111 | -0.042 | -0.004 | 0.039 |  |  |
| *Rothia* | 0.258 | -0.062 | -0.164 | 0.154 | 0.078 | 0.070 |  |  |
| *Veillonella* | -0.034 | 0.220 | 0.197 | -0.149 | 0.084 | -0.048 |  |  |

| *Prevotella* | -0.035 | 0.128 | 0.068 | -0.010 | -0.008 | 0.006 |  |  |
| --- | --- | --- | --- | --- | --- | --- | --- | --- |
| *Corynebacterium* | 0.000 | -0.095 | -0.150 | 0.110 | -0.062 | 0.001 |  |  |
| *Limosilactobacillus* | -0.149 | 0.174 | 0.225 | -0.208 | 0.038 | -0.094 |  |  |
| *Neisseria* | -0.037 | -0.124 | -0.114 | 0.032 | -0.022 | 0.027 |  |  |
| *Lactobacillus* | -0.175 | 0.137 | 0.169 | -0.204 | 0.071 | -0.052 |  |  |
| *Schaalia* | 0.083 | 0.032 | 0.078 | -0.093 | 0.129 | -0.012 |  |  |
| *Candida* | -0.073 | 0.269 | 0.305 | -0.268 | 0.051 | 0.079 |  |  |
| *Leptotrichia* | 0.017 | -0.038 | -0.080 | -0.087 | -0.033 | -0.053 |  |  |
| *Staphylococcus* | 0.032 | 0.064 | 0.006 | -0.063 | -0.037 | 0.163 |  |  |
| *Lacticaseibacillus* | -0.141 | 0.217 | 0.254 | -0.268 | 0.057 | -0.095 |  |  |
| *Parascardovia* | -0.064 | 0.195 | 0.216 | -0.192 | 0.085 | 0.082 |  |  |
| *Fusobacterium* | -0.087 | 0.054 | 0.068 | 0.032 | -0.047 | 0.054 |  |  |
| *Capnocytophaga* | -0.065 | 0.023 | 0.084 | -0.081 | 0.016 | -0.048 |  |  |
| *Gemella* | 0.099 | -0.117 | -0.133 | 0.305 | -0.012 | -0.078 |  |  |
| *Ligilactobacillus* | -0.126 | 0.146 | 0.164 | -0.297 | -0.040 | -0.092 |  |  |
| *Lactiplantibacillus* | -0.085 | 0.152 | 0.168 | -0.225 | -0.032 | -0.092 |  |  |
| **Species** | **Dentate/**  **Edentouolous** | **Age** | **Sex** | **Smoking** | **Diet** | **Diabetes** | **Dementia** | **Cognition** |
| *Rothia dentocariosa* | 0.186 | -0.037 | 0.081 | 0.085 | -0.003 | 0.052 | -0.134 | 0.021 |
| *Streptococcus oralis* | -0.002 | 0.012 | 0.041 | -0.088 | 0.082 | -0.005 | 0.210 | 0.323 |
| *Streptococcus gordonii* | 0.111 | -0.066 | 0.061 | 0.039 | -0.149 | -0.104 | 0.044 | -0.112 |
| *Veillonella parvula* | 0.252 | -0.112 | 0.106 | 0.198 | -0.166 | 0.067 | -0.026 | -0.134 |
| *Actinomyces oris* | 0.144 | 0.100 | 0.098 | 0.007 | -0.209 | 0.034 | -0.109 | -0.352 |
| *Limosilactobacillus fermentum* | 0.178 | -0.064 | -0.094 | -0.098 | 0.085 | 0.172 | -0.045 | 0.100 |
| *Corynebacterium matruchotii* | 0.065 | -0.093 | 0.145 | 0.156 | -0.119 | 0.033 | -0.073 | -0.254 |
| *Prevotella melaninogenica* | 0.072 | -0.081 | 0.035 | 0.226 | -0.013 | 0.009 | 0.196 | 0.075 |
| *Streptococcus parasanguinis* | 0.104 | -0.032 | -0.087 | -0.113 | 0.056 | 0.102 | 0.060 | 0.294 |

| *Streptococcus mutans* | 0.316 | 0.048 | -0.090 | 0.046 | -0.162 | 0.148 | -0.017 | -0.057 |
| --- | --- | --- | --- | --- | --- | --- | --- | --- |
| *Actinomyces sp. oral taxon 169* | 0.169 | 0.111 | 0.095 | 0.010 | -0.128 | 0.074 | -0.129 | -0.316 |
| *Streptococcus mitis* | -0.020 | -0.111 | -0.158 | -0.005 | 0.209 | 0.101 | 0.086 | 0.398 |
| *Schaalia odontolytica* | -0.028 | -0.195 | 0.002 | 0.269 | -0.159 | 0.035 | 0.166 | -0.067 |
| *Actinomyces radicidentis* | 0.111 | 0.023 | 0.063 | 0.236 | -0.013 | 0.079 | -0.179 | -0.185 |
| *Streptococcus vestibularis* | 0.107 | 0.024 | -0.148 | -0.026 | 0.097 | 0.120 | 0.074 | 0.272 |
| *Actinomyces sp. oral taxon 414* | 0.148 | -0.084 | 0.109 | 0.270 | -0.054 | 0.161 | -0.109 | -0.217 |
| *Streptococcus salivarius* | -0.079 | -0.067 | -0.153 | -0.080 | 0.115 | 0.088 | 0.150 | 0.355 |
| *Candida albicans* | 0.044 | -0.058 | -0.049 | 0.076 | 0.055 | 0.107 | -0.107 | 0.051 |
| *Streptococcus sobrinus* | 0.175 | 0.107 | 0.083 | -0.130 | -0.010 | -0.013 | 0.026 | 0.047 |
| *Actinomyces israelii* | 0.167 | 0.002 | 0.038 | 0.212 | -0.077 | 0.116 | -0.035 | -0.201 |
| **Species** | **Dry mouth** | **Oral mucosa** | **Denture stomatitis** | **Cleaning of denture** | **Denture type** | **Denture condition** |  |  |
| *Rothia dentocariosa* | 0.158 | -0.029 | -0.150 | 0.073 | 0.022 | 0.069 |  |  |
| *Streptococcus oralis* | 0.074 | -0.142 | -0.137 | 0.218 | -0.076 | -0.115 |  |  |
| *Streptococcus gordonii* | -0.015 | -0.170 | -0.195 | 0.015 | -0.006 | -0.098 |  |  |
| *Veillonella parvula* | -0.082 | 0.220 | 0.230 | -0.190 | 0.080 | -0.051 |  |  |
| *Actinomyces oris* | 0.062 | 0.100 | 0.090 | -0.168 | -0.019 | -0.050 |  |  |
| *Limosilactobacillus fermentum* | -0.130 | 0.194 | 0.256 | -0.233 | 0.041 | -0.089 |  |  |
| *Corynebacterium matruchotii* | -0.086 | -0.213 | -0.229 | 0.072 | -0.104 | 0.027 |  |  |
| *Prevotella melaninogenica* | 0.014 | 0.137 | 0.034 | 0.011 | -0.016 | 0.037 |  |  |
| *Streptococcus parasanguinis* | 0.193 | -0.089 | -0.067 | 0.120 | -0.030 | -0.210 |  |  |
| *Streptococcus mutans* | 0.012 | 0.129 | 0.128 | -0.182 | 0.078 | -0.027 |  |  |
| *Actinomyces sp. oral taxon 169* | 0.118 | 0.128 | 0.114 | -0.136 | 0.026 | -0.048 |  |  |
| *Streptococcus mitis* | 0.189 | -0.119 | -0.125 | 0.170 | -0.064 | -0.103 |  |  |
| *Schaalia odontolytica* | 0.082 | 0.028 | 0.074 | -0.098 | 0.124 | -0.016 |  |  |
| *Actinomyces radicidentis* | -0.049 | 0.174 | 0.105 | -0.055 | -0.023 | 0.054 |  |  |
| *Streptococcus vestibularis* | 0.038 | -0.020 | -0.079 | 0.068 | 0.015 | -0.145 |  |  |
| *Actinomyces sp. oral taxon 414* | -0.139 | 0.036 | 0.022 | -0.098 | 0.045 | 0.108 |  |  |
| *Streptococcus salivarius* | 0.127 | -0.083 | -0.121 | 0.181 | -0.012 | -0.057 |  |  |
| *Candida albicans* | -0.109 | 0.267 | 0.328 | -0.278 | 0.062 | 0.063 |  |  |
| *Streptococcus sobrinus* | -0.058 | 0.104 | 0.086 | -0.032 | -0.072 | -0.043 |  |  |
| *Actinomyces israelii* | -0.087 | 0.118 | 0.079 | -0.080 | 0.018 | 0.036 |  |  |

50

51

52 **Table S6.** Spearman correlation coefficients between the top most abundant genera and species and clinical/oral health variables in dentate 53 participants.

| **Genus/Species** | **Total no of teeth** | **Mobile teeth** | **Periodontitis** | **BOP teeth** | **PPD**  **teeth 4-**  **5mm** | **PPD**  **teeth 4-**  **5mm** | **Plaque Index** | **Gingival Index** | **Root caries teeth** | **Crown caries teeth** |
| --- | --- | --- | --- | --- | --- | --- | --- | --- | --- | --- |
| *Streptococcus* | -0.044 | 0.178 | 0.097 | -0.056 | 0.344 | 0.122 | -0.111 | -0.083 | -0.057 | -0.188 |
| *Actinomyces* | 0.048 | -0.138 | 0.051 | -0.006 | -0.175 | 0.068 | -0.106 | 0.003 | 0.137 | 0.018 |
| *Rothia* | -0.178 | 0.022 | 0.115 | -0.346 | 0.160 | -0.011 | -0.149 | -0.063 | 0.084 | -0.245 |
| *Veillonella* | -0.061 | 0.128 | -0.036 | 0.025 | -0.053 | -0.112 | 0.053 | 0.024 | -0.282 | 0.216 |
| *Prevotella* | 0.131 | 0.074 | -0.138 | 0.446 | -0.107 | 0.058 | 0.338 | 0.325 | -0.044 | 0.081 |
| *Corynebacterium* | 0.005 | 0.012 | 0.083 | 0.080 | -0.111 | 0.235 | 0.074 | 0.127 | 0.366 | -0.120 |
| *Limosilactobacillus* | -0.003 | -0.014 | -0.061 | 0.060 | -0.187 | 0.049 | 0.121 | 0.010 | -0.001 | 0.125 |
| *Lactobacillus* | -0.076 | 0.051 | -0.105 | 0.061 | -0.031 | -0.013 | 0.224 | -0.011 | -0.078 | 0.135 |
| *Neisseria* | -0.065 | 0.130 | 0.253 | 0.064 | 0.228 | 0.188 | 0.015 | 0.153 | 0.014 | -0.101 |
| *Parascardovia* | 0.020 | -0.245 | -0.241 | 0.130 | -0.372 | -0.140 | 0.046 | 0.103 | -0.012 | 0.143 |
| *Candida* | -0.083 | -0.073 | 0.022 | -0.085 | -0.112 | 0.134 | -0.025 | -0.129 | -0.102 | -0.070 |
| *Schaalia* | -0.079 | 0.092 | 0.167 | 0.146 | 0.070 | 0.062 | 0.227 | 0.334 | -0.119 | -0.062 |
| *Lacticaseibacillus* | -0.093 | 0.114 | 0.113 | 0.014 | 0.034 | 0.064 | 0.027 | -0.211 | -0.057 | 0.132 |
| *Leptotrichia* | -0.108 | -0.003 | 0.130 | 0.047 | 0.066 | 0.018 | -0.118 | 0.157 | 0.035 | -0.220 |
| *Ligilactobacillus* | 0.037 | -0.081 | -0.021 | 0.072 | -0.007 | 0.012 | 0.023 | -0.088 | -0.042 | 0.044 |
| *Fusobacterium* | 0.188 | 0.093 | -0.056 | 0.336 | -0.046 | 0.006 | 0.192 | 0.339 | 0.002 | -0.126 |
| *Lancefieldella* | 0.083 | 0.038 | -0.029 | 0.269 | -0.071 | 0.094 | 0.382 | 0.169 | -0.120 | 0.297 |
| *Gemella* | 0.079 | 0.346 | 0.117 | 0.372 | 0.275 | -0.005 | 0.281 | 0.336 | 0.006 | -0.190 |
| *Capnocytophaga* | -0.011 | 0.080 | 0.090 | 0.028 | 0.071 | -0.020 | 0.016 | 0.262 | -0.009 | -0.185 |
| *Propionibacterium* | 0.243 | -0.182 | -0.059 | 0.333 | 0.004 | -0.037 | 0.277 | 0.025 | -0.002 | 0.073 |
| *Rothia dentocariosa* | -0.125 | -0.108 | -0.018 | -0.378 | 0.040 | -0.103 | -0.222 | -0.167 | 0.024 | -0.187 |
| *Veillonella parvula* | -0.067 | 0.143 | -0.047 | 0.001 | -0.065 | -0.151 | 0.001 | 0.057 | -0.335 | 0.163 |
| *Streptococcus gordonii* | 0.038 | -0.010 | -0.160 | -0.147 | 0.110 | -0.260 | -0.043 | -0.074 | -0.300 | -0.243 |
| *Streptococcus oralis* | 0.042 | 0.094 | -0.036 | 0.159 | 0.206 | 0.084 | 0.033 | 0.054 | -0.105 | -0.369 |
| *Actinomyces oris* | -0.015 | -0.266 | -0.116 | -0.032 | -0.096 | -0.185 | -0.082 | -0.108 | 0.033 | -0.038 |
| *Streptococcus mutans* | -0.159 | -0.187 | -0.262 | -0.109 | -0.304 | -0.150 | 0.119 | 0.052 | -0.054 | 0.007 |
| *Limosilactobacillus fermentum* | -0.008 | -0.038 | -0.052 | 0.043 | -0.195 | 0.074 | 0.100 | 0.006 | -0.007 | 0.133 |
| *Prevotella melaninogenica* | 0.061 | 0.101 | -0.096 | 0.409 | -0.007 | -0.017 | 0.309 | 0.267 | -0.003 | 0.133 |
| *Streptococcus parasanguinis* | -0.133 | 0.193 | 0.201 | -0.228 | 0.197 | 0.193 | -0.269 | -0.286 | -0.056 | -0.043 |
| *Streptococcus sobrinus* | 0.039 | 0.049 | -0.057 | -0.038 | 0.020 | -0.043 | -0.018 | -0.291 | -0.051 | 0.026 |
| *Actinomyces sp. oral taxon 414* | -0.015 | -0.191 | -0.037 | 0.016 | -0.140 | -0.009 | 0.044 | 0.172 | -0.029 | 0.048 |
| *Actinomyces israelii* | 0.079 | -0.152 | -0.049 | 0.125 | -0.242 | -0.072 | 0.134 | 0.236 | -0.006 | -0.086 |
| *Parascardovia denticolens* | 0.020 | -0.245 | -0.241 | 0.130 | -0.372 | -0.140 | 0.046 | 0.103 | -0.012 | 0.143 |
| *Corynebacterium matruchotii* | 0.032 | -0.184 | -0.139 | 0.097 | -0.193 | 0.113 | 0.077 | 0.169 | 0.202 | -0.105 |
| *Streptococcus vestibularis* | 0.041 | 0.214 | -0.025 | 0.035 | 0.072 | 0.130 | -0.004 | 0.071 | -0.077 | -0.098 |
| *Corynebacterium pseudokroppenstedtii* | 0.089 | -0.076 | 0.029 | 0.022 | -0.151 | 0.146 | 0.016 | -0.107 | 0.167 | -0.093 |
| *Actinomyces sp. oral taxon 169* | -0.197 | -0.188 | -0.087 | -0.256 | -0.095 | -0.178 | -0.178 | -0.175 | 0.009 | -0.034 |
| *Schaalia odontolytica* | -0.064 | 0.083 | 0.176 | 0.148 | 0.085 | 0.065 | 0.215 | 0.344 | -0.125 | -0.059 |
| *Actinomyces radicidentis* | 0.076 | 0.103 | 0.225 | 0.146 | 0.002 | 0.149 | 0.074 | 0.063 | 0.145 | 0.076 |
| *Candida albicans* | -0.189 | 0.033 | 0.124 | -0.149 | -0.016 | 0.169 | -0.113 | -0.175 | -0.153 | -0.059 |

54

55

56 **Table S7.** Network topography metrics and hub genera for the top oral genera in edentulous 57 and dentate participants.

|  | **Edentulous** | **Dentate** |
| --- | --- | --- |
| Edges | 13 | 28 |
| Avg Degree | 0.53 | 1.14 |
| Density | 0.0111 | 0.0238 |
| Max Degree | 3 | 5 |
| Avg Clustering | 0.2381 | 0.2833 |
| Hub Genera | *Micrococcus, Acinetobacter,*  *Limosilactobacillus,*  *Cutibacterium, Pseudomonas* | *Eikenella, Neisseria,*  *Ligilactobacillus,*  *Limosilactobacillus, Selenomonas* |

58

1. **Table S8. Functional pathway differences between edentulous and dentate DAOM**

| **MetaCycID** | **Category** | **Pathway** | **Species** | **Genus** | **Coef** | **SE** | ***P*** |
| --- | --- | --- | --- | --- | --- | --- | --- |
| ANAGLYCOLYS  IS-PWY | Carbohydrate Metabolism | Glycolysis III (from glucose) | *Streptococcus mitis* | *Streptococcus* | -9.075 | 4.571 | 0.049 |
| ARO.PWY | Amino Acid Metabolism | Chorismate Biosynthesis I | *Cryptobacterium curtum* | *Cryptobacterium* | 0.508 | 0.220 | 0.022 |
| BRANCHED-  CHAIN-AA-SYNPWY | Amino Acid Metabolism | Superpathway of Branched-  Chain Amino Acid Biosynthesis | *Streptococcus pneumoniae* | *Streptococcus* | -12.368 | 5.500 | 0.026 |
| CALVIN.PWY | Energy  Metabolism | Calvin–Benson–Bassham Cycle | *Lachnoanaerobaculum saburreum* | *Lachnoanaerobacul um* | -0.973 | 0.463 | 0.038 |
| COA.PWY | Vitamin &  Cofactor  Metabolism | Coenzyme A Biosynthesis I (prokaryotic) | *Actinomyces hongkongensis* | *Actinomyces* | -1.561 | 0.707 | 0.029 |
| DTDPRHAMSYN  .PWY | Carbohydrate Metabolism | dTDP-L-rhamnose Biosynthesis | *Lactobacillus oris* | *Lactobacillus* | 0.493 | 0.238 | 0.041 |
| GLYCOGENSYN  TH.PWY | Carbohydrate Metabolism | Glycogen Biosynthesis I (from ADP-D-Glucose) | *Streptococcus pneumoniae* | *Streptococcus* | -12.040 | 5.408 | 0.028 |
| HEMESYN2.PW  Y | Vitamin &  Cofactor  Metabolism | Heme B Biosynthesis II (oxygen-independent) | *Neisseria sp oral taxon 014* | *Neisseria* | 0.174 | 0.085 | 0.043 |
| HSERMETANA.P WY | Amino Acid Metabolism | L-Methionine Biosynthesis III | *Streptococcus pneumoniae* | *Streptococcus* | -12.443 | 4.832 | 0.011 |
| ILEUSYN.PWY | Amino Acid Metabolism | L-Isoleucine Biosynthesis I (from threonine) | *Streptococcus pneumoniae* | *Streptococcus* | -11.880 | 5.473 | 0.032 |
| LACTOSECAT.P WY | Carbohydrate Metabolism | Lactose and Galactose Degradation I | *Streptococcus oralis* | *Streptococcus* | -88.511 | 43.364 | 0.043 |
| NONMEVIPP.PW  Y | Vitamin &  Cofactor  Metabolism | Methylerythritol Phosphate (MEP) Pathway I | *Cryptobacterium curtum* | *Cryptobacterium* | 0.437 | 0.203 | 0.034 |

| NONOXIPENT.P WY | Carbohydrate Metabolism | Pentose Phosphate Pathway, Non-Oxidative Branch I | *Actinomyces hongkongensis* | *Actinomyces* | -2.026 | 0.941 | 0.033 |
| --- | --- | --- | --- | --- | --- | --- | --- |
| OANTIGEN.PWY | Carbohydrate Metabolism | O-Antigen Building Blocks Biosynthesis (E. coli) | *Fusobacterium nucleatum* | *Fusobacterium* | 1.980 | 0.904 | 0.030 |
| P161.PWY | Energy  Metabolism | Acetylene Degradation (Anaerobic) | *Streptococcus oralis* | *Streptococcus* | -18.377 | 8.837 | 0.040 |
| P41.PWY | Energy  Metabolism | Pyruvate Fermentation to Acetate and L-Lactate | *Lachnoanaerobaculum saburreum* | *Lachnoanaerobacul um* | -0.933 | 0.470 | 0.049 |
| PENTOSE.P.PWY | Carbohydrate Metabolism | Pentose Phosphate Pathway | *Streptococcus pneumoniae* | *Streptococcus* | -2.914 | 1.217 | 0.018 |
| PEPTIDOGLYCA NSYN.PWY | Carbohydrate Metabolism | Peptidoglycan Biosynthesis I (meso-diaminopimelate containing) | *Cryptobacterium curtum* | *Cryptobacterium* | 0.487 | 0.204 | 0.019 |
| PWY.1042 | Carbohydrate Metabolism | Glycolysis IV | *Streptococcus oralis* | *Streptococcus* | -27.002 | 13.038 | 0.040 |
| PWY.2941 | Amino Acid Metabolism | L-Lysine Biosynthesis II | *Lactobacillus rhamnosus* | *Lactobacillus* | 1.088 | 0.453 | 0.018 |
| PWY.3001 | Amino Acid Metabolism | Superpathway of L-Isoleucine Biosynthesis I | *Streptococcus pneumoniae* | *Streptococcus* | -9.358 | 4.398 | 0.035 |
| PWY.5097 | Amino Acid Metabolism | L-Lysine Biosynthesis VI | *Actinomyces hongkongensis* | *Actinomyces* | -1.805 | 0.800 | 0.026 |
| PWY.5100 | Energy  Metabolism | Pyruvate Fermentation to Acetate and Lactate II | *Lachnoanaerobaculum saburreum* | *Lachnoanaerobacul um* | -0.933 | 0.470 | 0.049 |
| PWY.5103 | Amino Acid Metabolism | L-Isoleucine Biosynthesis III | *Streptococcus pneumoniae* | *Streptococcus* | -9.914 | 4.775 | 0.040 |
| PWY.5367 | Lipid & Fatty  Acid  Metabolism | Petroselinate Biosynthesis | *Streptococcus parasanguinis* | *Streptococcus* | -2.003 | 1.001 | 0.047 |
| PWY.5384 | Carbohydrate Metabolism | Sucrose Degradation IV (sucrose phosphorylase) | *Lactobacillus oris* | *Lactobacillus* | 1.080 | 0.462 | 0.021 |
| PWY.5659 | Carbohydrate Metabolism | GDP-Mannose Biosynthesis | *Streptococcus pneumoniae* | *Streptococcus* | -9.357 | 4.229 | 0.029 |

| PWY.5667 | Lipid & Fatty  Acid  Metabolism | CDP-Diacylglycerol Biosynthesis I | *Lactobacillus oris* | *Lactobacillus* | 0.741 | 0.362 | 0.043 |
| --- | --- | --- | --- | --- | --- | --- | --- |
| PWY.5686 | Nucleotide Metabolism | UMP Biosynthesis I | *Actinomyces hongkongensis* | *Actinomyces* | -2.020 | 0.917 | 0.029 |
| PWY.5941 | Carbohydrate Metabolism | Glycogen Degradation II | *Streptococcus pneumoniae* | *Streptococcus* | -18.483 | 7.276 | 0.012 |
| PWY.5973 | Lipid & Fatty  Acid  Metabolism | cis-Vaccenate Biosynthesis | *Lactobacillus oris* | *Lactobacillus* | 4.279 | 1.991 | 0.034 |
| PWY.5981 | Lipid & Fatty  Acid  Metabolism | CDP-Diacylglycerol Biosynthesis III | *Lactobacillus rhamnosus* | *Lactobacillus* | 0.725 | 0.361 | 0.047 |
| PWY.6121 | Nucleotide Metabolism | 5-Aminoimidazole  Ribonucleotide Biosynthesis I | *Cryptobacterium curtum* | *Cryptobacterium* | 0.438 | 0.185 | 0.019 |
| PWY.6122 | Nucleotide Metabolism | 5-Aminoimidazole  Ribonucleotide Biosynthesis II | *Actinomyces hongkongensis* | *Actinomyces* | -2.271 | 1.023 | 0.028 |
| PWY.6123 | Nucleotide Metabolism | Inosine 5′-Phosphate Biosynthesis I | *Streptococcus mitis* | *Streptococcus* | -24.59 | 10.53 | 0.021 |
| PWY.6124 | Nucleotide Metabolism | Inosine 5′-Phosphate Biosynthesis II | *Streptococcus mitis* | *Streptococcus* | -33.014 | 12.808 | 0.011 |
| PWY.6125 | Nucleotide Metabolism | Superpathway of Guanosine  Nucleotides de novo Biosynthesis II | *Streptococcus salivarius* | *Streptococcus* | -13.492 | 6.690 | 0.046 |
| PWY.6147 | Vitamin &  Cofactor  Metabolism | 6-Hydroxymethyl Dihydropterin  Diphosphate Biosynthesis I | *Streptococcus gordonii* | *Streptococcus* | 1.675 | 0.718 | 0.021 |
| PWY.6151 | Vitamin &  Cofactor  Metabolism | S-Adenosyl-L-Methionine Salvage I | *Cryptobacterium curtum* | *Cryptobacterium* | 0.421 | 0.169 | 0.014 |
| PWY.6163 | Amino Acid Metabolism | Chorismate Biosynthesis from 3Dehydroquinate | *Veillonella rogosae* | *Veillonella* | -0.063 | 0.029 | 0.036 |

| PWY.621 | Carbohydrate Metabolism | Sucrose Degradation III (sucrose invertase) | *Lactobacillus oris* | *Lactobacillus* | 0.904 | 0.409 | 0.029 |
| --- | --- | --- | --- | --- | --- | --- | --- |
| PWY.6277 | Nucleotide Metabolism | Superpathway of 5-  Aminoimidazole Ribonucleotide Biosynthesis | *Streptococcus pneumoniae* | *Streptococcus* | -10.859 | 4.662 | 0.021 |
| PWY.6317 | Carbohydrate Metabolism | D-Galactose Degradation I (Leloir Pathway) | *Streptococcus mitis* | *Streptococcus* | -18.992 | 7.967 | 0.018 |
| PWY.6385 | Carbohydrate Metabolism | Peptidoglycan Biosynthesis III (Mycobacteria) | *Cryptobacterium curtum* | *Cryptobacterium* | 0.470 | 0.198 | 0.019 |
| PWY.6386 | Carbohydrate Metabolism | UDP-N-Acetylmuramoyl-  Pentapeptide Biosynthesis II (Lysine-containing) | *Cryptobacterium curtum* | *Cryptobacterium* | 0.480 | 0.201 | 0.019 |
| PWY.6387 | Carbohydrate Metabolism | UDP-N-AcetylmuramoylPentapeptide Biosynthesis I  (meso-Diaminopimelate) | *Cryptobacterium curtum* | *Cryptobacterium* | 0.491 | 0.207 | 0.019 |
| PWY.6527 | Carbohydrate Metabolism | Stachyose Degradation | *Streptococcus oralis* | *Streptococcus* | -50.12 | 24.746 | 0.045 |
| PWY.6609 | Nucleotide Metabolism | Adenine and Adenosine Salvage  III | *Streptococcus pneumoniae* | *Streptococcus* | -14.54 | 7.077 | 0.042 |
| PWY.6628 | Amino Acid Metabolism | Superpathway of L-  Phenylalanine Biosynthesis | *Lachnoanaerobaculum saburreum* | *Lachnoanaerobacul um* | -0.446 | 0.215 | 0.041 |
| PWY.6700 | Vitamin &  Cofactor  Metabolism | Queuosine Biosynthesis I (de novo) | *Streptococcus salivarius* | *Streptococcus* | -11.029 | 5.477 | 0.046 |
| PWY.6703 | Vitamin &  Cofactor  Metabolism | preQ₀ Biosynthesis | *Streptococcus mitis* | *Streptococcus* | -12.029 | 6.053 | 0.049 |
| PWY.6823 | Vitamin &  Cofactor  Metabolism | Molybdopterin Biosynthesis | *Cryptobacterium curtum* | *Cryptobacterium* | 0.486 | 0.210 | 0.022 |

| PWY.6936 | Vitamin &  Cofactor  Metabolism | Seleno-Amino Acid Biosynthesis (Plants) | *Streptococcus oralis* | *Streptococcus* | -50.15 | 23.35 | 0.034 |
| --- | --- | --- | --- | --- | --- | --- | --- |
| PWY.702 | Amino Acid Metabolism | L-Methionine Biosynthesis II | *Streptococcus pneumoniae* | *Streptococcus* | -14.813 | 6.988 | 0.036 |
| PWY.7111 | Energy  Metabolism | Pyruvate Fermentation to Isobutanol (Engineered) | *Streptococcus pneumoniae* | *Streptococcus* | -10.69 | 5.280 | 0.045 |
| PWY.7185 | Nucleotide Metabolism | UTP and CTP  Dephosphorylation I | *Lactobacillus rhamnosus* | *Lactobacillus* | 1.992 | 0.937 | 0.035 |
| PWY.7197 | Nucleotide Metabolism | Pyrimidine Deoxyribonucleotide Phosphorylation | *Cryptobacterium curtum* | *Cryptobacterium* | 0.427 | 0.183 | 0.021 |
| PWY.7208 | Nucleotide Metabolism | Superpathway of Pyrimidine Nucleobases Salvage | *Streptococcus mitis* | *Streptococcus* | -22.67 | 11.25 | 0.046 |
| PWY.7220 | Nucleotide Metabolism | Adenosine  Deoxyribonucleotides de novo Biosynthesis II | *Streptococcus mitis* | *Streptococcus* | -31.24 | 14.71 | 0.036 |
| PWY.7221 | Nucleotide Metabolism | Guanosine Ribonucleotides de novo Biosynthesis | *Streptococcus anginosus group* | *Streptococcus* | 0.421 | 0.206 | 0.043 |
| PWY.7222 | Nucleotide Metabolism | Guanosine deoxyribonucleotides de novo biosynthesis II | *Streptococcus mitis* | *Streptococcus* | -31.24 | 14.71 | 0.036 |
| PWY.7228. | Nucleotide Metabolism | Superpathway of Guanosine  Nucleotides de novo Biosynthesis I | *Streptococcus salivarius* | *Streptococcus* | -15.42 | 7.698 | 0.047 |
| PWY.7234 | Nucleotide Metabolism | Inosine 5′-Phosphate Biosynthesis III | *Lactobacillus rhamnosus* | *Lactobacillus* | 0.838 | 0.421 | 0.049 |
| PWY.7238 | Carbohydrate Metabolism | Sucrose Biosynthesis II | *Streptococcus pneumoniae* | *Streptococcus* | -16.654 | 6.744 | 0.015 |
| PWY.7282 | Vitamin &  Cofactor  Metabolism | 4-Amino-2-Methyl-5Diphosphomethylpyrimidine Biosynthesis II | *Streptococcus pneumoniae* | *Streptococcus* | -1.403 | 0.665 | 0.037 |

| PWY.7356 | Vitamin &  Cofactor  Metabolism | Thiamine Diphosphate Salvage IV (Yeast) | *Lactobacillus rhamnosus* | *Lactobacillus* | 0.706 | 0.330 | 0.034 |
| --- | --- | --- | --- | --- | --- | --- | --- |
| PWY.7357 | Vitamin &  Cofactor  Metabolism | Thiamine Phosphate Formation from Pyrithiamine and Oxythiamine (Yeast) | *Lachnoanaerobaculum saburreum* | *Lachnoanaerobacul um* | -0.501 | 0.220 | 0.025 |
| PWY.7663 | Lipid & Fatty  Acid  Metabolism | Gondoate Biosynthesis (Anaerobic) | *Lactobacillus oris* | *Lactobacillus* | 4.977 | 2.191 | 0.025 |
| PWY.7790 | Nucleotide Metabolism | UMP Biosynthesis II | *Streptococcus pneumoniae* | *Streptococcus* | -11.16 | 5.604 | 0.049 |
| PWY.7953 | Carbohydrate Metabolism | UDP-N-Acetylmuramoyl-  Pentapeptide Biosynthesis III (meso-Diaminopimelatecontaining) | *Cryptobacterium curtum* | *Cryptobacterium* | 0.488 | 0.204 | 0.018 |
| PWY.8178 | Carbohydrate Metabolism | Pentose Phosphate Pathway, Non-Oxidative Branch II | *Streptococcus pneumoniae* | *Streptococcus* | -9.007 | 4.533 | 0.049 |
| PWY0.1296 | Nucleotide Metabolism | Purine Ribonucleosides Degradation | *Streptococcus pneumoniae* | *Streptococcus* | -16.05 | 7.110 | 0.026 |
| PWY0.1297 | Nucleotide Metabolism | Superpathway of Purine  Deoxyribonucleosides  Degradation (Streptococcus) | *Streptococcus oralis* | *Streptococcus* | -16.78 | 8.313 | 0.046 |
| PWY0.1319 | Lipid & Fatty  Acid  Metabolism | CDP-Diacylglycerol Biosynthesis II | *Lactobacillus oris* | *Lactobacillus* | 0.741 | 0.362 | 0.043 |
| PWY0.1477 | Amino Acid Metabolism | Ethanolamine Utilization | *Streptococcus oralis* | *Streptococcus* | -18.37 | 8.837 | 0.040 |
| PWY0.1586 | Carbohydrate Metabolism | Peptidoglycan Maturation (meso-Diaminopimelatecontaining) | *Neisseria sp_oral_taxon_014* | *Neisseria* | 0.398 | 0.191 | 0.040 |
| PWY0.301 | Carbohydrate Metabolism | L-Ascorbate Degradation I (Bacterial, Anaerobic) | *Lactobacillus rhamnosus* | *Lactobacillus* | 1.417 | 0.703 | 0.046 |
| PWY66.409 | Nucleotide Metabolism | Superpathway of Purine Nucleotide Salvage | *Streptococcus pneumoniae* | *Streptococcus* | -3.276 | 1.577 | 0.040 |
| RHAMCAT.PWY | Carbohydrate Metabolism | L-Rhamnose Degradation I | *Lactobacillus salivarius* | *Lactobacillus* | 2.812 | 1.248 | 0.026 |
| SER.GLYSYN.P WY | Amino Acid Metabolism | Superpathway of L-Serine and Glycine Biosynthesis I | *Actinomyces hongkongensis* | *Actinomyces* | -1.957 | 0.863 | 0.025 |
| THRESYN.PWY | Amino Acid Metabolism | Superpathway of L-Threonine Biosynthesis | *Streptococcus pneumoniae* | *Streptococcus* | -17.81 | 8.878 | 0.047 |
| TRNA.CHARGIN G.PWYae | Amino Acid Metabolism | tRNA Charging | *Streptococcus pneumoniae* | *Streptococcus* | -9.602 | 4.596 | 0.039 |
| UDPNAGSYN.P WY | Carbohydrate Metabolism | UDP-N-Acetyl-D-Glucosamine  Biosynthesis I | *Streptococcus troglodytae* | *Streptococcus* | 0.018 | 0.007 | 0.023 |
| VALSYN.PWY | Amino Acid Metabolism | L-Valine Biosynthesis | *Streptococcus pneumoniae* | *Streptococcus* | -16.551 | 6.795 | 0.016 |

1. Coefficients represent associations with edentulism status (negative = higher in edentulous participants, positive = higher in dentate participants). 61 All *q*-values = 0.567.
